# CD73: agent development potential and its application in diabetes and atherosclerosis

**DOI:** 10.3389/fimmu.2024.1515875

**Published:** 2024-12-12

**Authors:** Dan Liu, Jingjing Zhao, Ling Li, Jie Wang, Chao Wang, Yudong Wu, Yucun Huang, Dongming Xing, Wujun Chen

**Affiliations:** ^1^ Guangdong Provincial People’s Hospital, Zhuhai Hospital (Jinwan Central Hospital of Zhuhai), Zhuhai, Guangdong, China; ^2^ Sleep Medicine Center, Huai’an No.3 People’s Hospital, Huaian Second Clinical College of Xuzhou Medical University, Huaian, China; ^3^ Department of Pharmacy, The Fifth Affiliated Hospital, Sun Yat-sen University, Zhuhai, Guangdong, China; ^4^ The Affiliated Hospital of Qingdao University, Qingdao University, Qingdao Cancer Institute, Qingdao, Shandong, China; ^5^ School of Life Sciences, Tsinghua University, Beijing, China

**Keywords:** CD73, circNT5E, diabetes, atherosclerosis, agent development

## Abstract

CD73, an important metabolic and immune escape-promoting gene, catalyzes the hydrolysis of adenosine monophosphate (AMP) to adenosine (ADO). AMP has anti-inflammatory and vascular relaxant properties, while ADO has a strong immunosuppressive effect, suggesting that CD73 has pro-inflammatory and immune escape effects. However, CD73 also decreased proinflammatory reaction, suggesting that CD73 has a positive side to the body. Indeed, CD73 plays a protective role in diabetes, while with age, CD73 changes from anti-atherosclerosis to pro-atherosclerosis. The upregulation of CD73 with agents, including AGT-5, Aire-overexpressing DCs, Aspirin, BAFFR-Fc, CD4+ peptide, ICAs, IL-2 therapies, SAgAs, sCD73, stem cells, RAD51 inhibitor, TLR9 inhibitor, and VD, decreased diabetes and atherosclerosis development. However, the downregulation of CD73 with agents, including benzothiadiazine derivatives and CD73 siRNA, reduced atherosclerosis. Notably, many CD73 agents were investigated in clinical trials. However, no agents were used to treat diabetes and atherosclerosis. Most agents were CD73 inhibitors. Only FP-1201, a CD73 agonist, was investigated in clinical trials but its further development was discontinued. In addition, many lncRNAs, circRNAs, and genes are located at the same chromosomal location as CD73. In particular, circNT5E promoted CD73 expression. circNT5E may be a promising target for agent development. This mini-review focuses on the current state of knowledge of CD73 in diabetes, atherosclerosis, and its potential role in agent development.

## 1 Introduction

CD73 [also named ecto-5’-nucleotidase (5NTE), a cell surface-bound nucleotidase, is an important metabolic and immune escape-promoting gene. CD73 catalyzes the hydrolysis of adenosine monophosphate (AMP) to adenosine (ADO). AMP has anti-inflammatory and vascular relaxant properties, while ADO has a strong immunosuppressive effect by adenosine A2A receptor (A2AR) and A2BR, suggesting that CD73 promotes tumor cells to achieve immune escape (1–3). However, CD73 also has a positive side to the body. CD73 decreased proinflammatory reaction by promoting M2 macrophage phenotype (anti-inflammatory), enhancing endothelial barrier function, and inhibiting leukocyte trafficking (4). CD73 is a mesenchymal stem cell (MSC) and Breg-specific marker (5–8). CD73 is associated with a variety of diseases, including atherosclerosis, cancer, cirrhotic cardiomyopathy, diabetes, graft-versus-host disease (GVHD), periodontitis, rheumatoid arthritis, and systemic lupus erythematosus (SLE) (1–3, 9–12). Especially in cancer, the role and mechanism of CD73 have been reviewed and studied by multiple laboratories (1–3). However, few reviews exist on the agent development of CD73 and its role in diabetes and atherosclerosis. The main aims of this mini-review are to describe the current state of knowledge of CD73 in diabetes, atherosclerosis, and its potential role in agent development.

## 2 The role and mechanism of CD73 in diabetes and atherosclerosis

### 2.1 Diabetes

**Figure 1:**
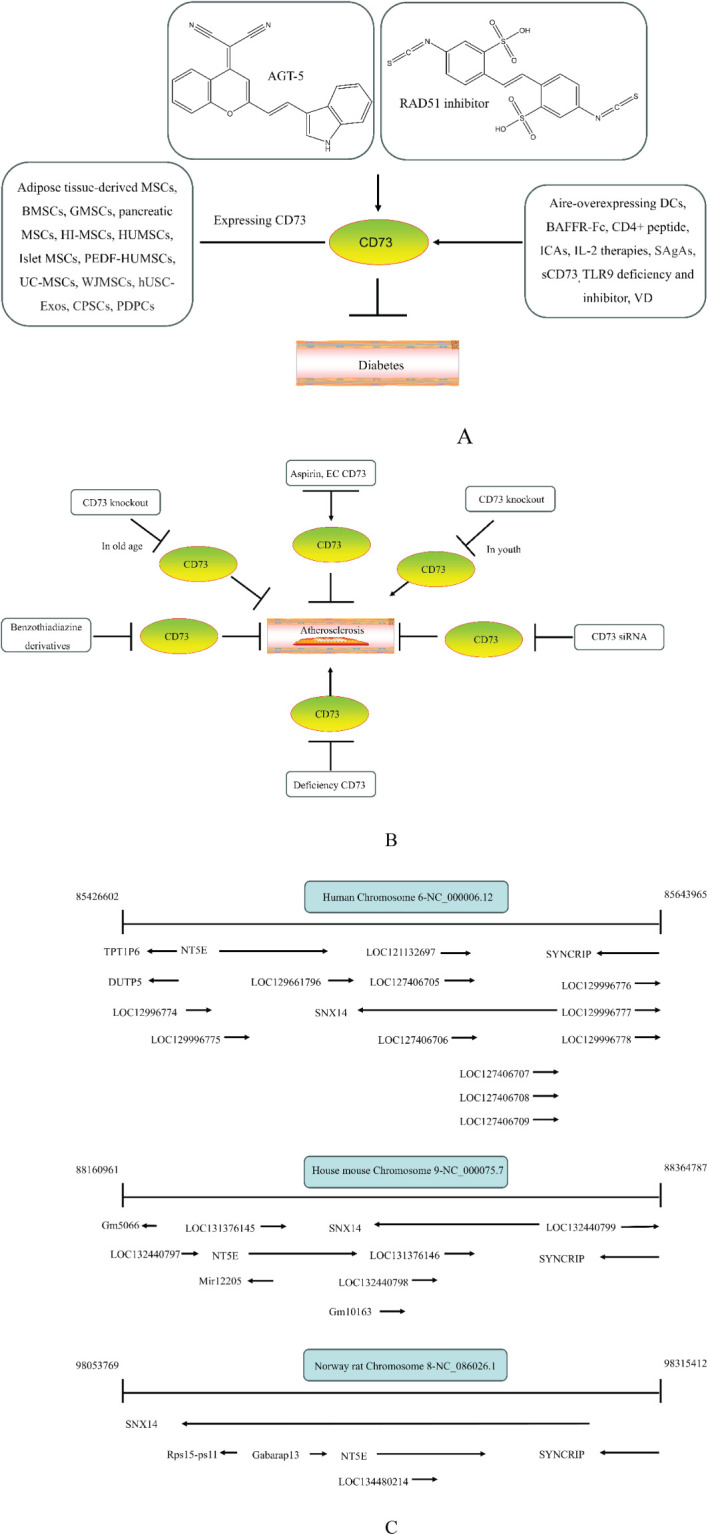
The role of CD73 agents in diabetes and atherosclerosis and the lncRNAs and genes at the same chromosomal location as CD73. **(A)** Diabetes, **(B)** atherosclerosis, **(C)** This information was modified from gene (NIH).

CD73 was increased in the kidneys of diabetic mice. The absence of CD73 was positively associated with the severity of diabetic nephropathy, suggesting that CD73 is a potential biomarker of diabetic nephropathy (13). B lymphocytes promote the development of type 1 diabetes mellitus (T1DM) by promoting the expansion of pathogenic T cells. Anti-CD20, a B lymphocyte-targeted therapy, promoted B lymphocyte depletion. However, they failed to halt β cell demise. Suppressing RAD51 with CRISPR/cas9 and inhibitors (such as 4,4’-diisothiocyanatostilbene-2, 2’-disulfonic acid) decreases the diabetes process by reducing diabetogenic T cell responses via expanding CD73+ B lymphocytes that exert regulatory activity in T1DM-susceptible nonobese diabetic (NOD) mice (14). Soluble BAFF receptor (BAFFR)-Fc (BAFFR-Fc), a fusion protein that fuses with the extracellular part of BAFFR to the Fc domain of mouse IgG1, was developed by MedImmune. BAFFR-Fc decreases T1DM procession by expanding CD73+ B lymphocytes and reduces side effects of anti-CD20 (15), suggesting that CD73 plays a key role in reducing side effects of B lymphocyte-targeted therapies. Indeed, many studies have shown that CD73 plays a protective role in diabetes. For example, AGT-5 (oral compound), a new class of fluorescent aryl hydrocarbon receptor (AHR) ligands (FluoAHRL), suppressed the severity of streptozotocin (STZ)-induced T1DM by enhancing AHR and CD73 expression in mice (16). Vitamin D3 (VD, 25-(OH)D3) reduced diabetes mellitus (DM)-related cognitive dysfunction by enhancing CD39 and CD73 expression in streptozotocin-induced T1DM rats (17). Autoimmune regulator (Aire)-overexpressing dendritic cells (DCs) delayed T1DM processing by reducing CD4^+^ IFN-γ^+^ T cells level and enhancing CD73, lymphocyte activation Gene-3 (LAG-3), and folic acid receptor 4 (FR4) expression and CD4^+^ T cells apoptosis in splenocytes in STZ-T1DM mouse (18). The islet-like cell aggregates (ICAs) decreased diabetes procession by expressing CD73 in STZ-induced diabetic mice (19). Interleukin-2 (IL-2) therapies with anti-IL-2 antibodies decrease the T1DM process by enhancing CD25, CD39, and CD73 expression in regulatory T cells (Treg cells) in NOD mice (20). Liposomes encapsulating the CD4+ peptide [BDC2.5mim, has a high affinity for islet autoantigen chromogranin a (ChgA)] and 1α,25-dihydroxy vitamin D3 (calcitriol) suppressed diabetes progression by activating ChgA-specific forkhead box P3 (Foxp3)+ and Foxp3- programmed cell death 1 (PD1)+ CD73+ inducible T cell costimulator (ICOS, also named CD278)+ IL-10+ peripheral regulatory T cells (21), suggesting that CD4+ peptide combination with calcitriol decreased diabetes progression by enhancing CD73+ expression. Soluble CD73 (sCD73) decreased diabetic nephropathy (13). Soluble antigen arrays (SAgAs) were able to bind more effectively to antigen-specific T cells, such as CD73, IL-10, PD-1, and killer cell lectin-like receptor G1 (KLRG1), alleviating disease progression in non-obese diabetic mouse models of T1DM (22). Toll-like receptor 9 (TLR9) deficiency and inhibitor decreases diabetes development by reducing proinflammatory cytokines and promoting anti-inflammatory cytokine release via enhancing CD73 expression in T cells in NOD mice. The increase in CD73-expressing immune cells is specific for TLR9 deficiency (23), suggesting that CD73 plays a key role in TLR9 inhibitors in reducing diabetes development. In summary, CD73 may be a promising target for treating diabetes. Overexpressing CD73 with agents, such as AGT-5, Aire-overexpressing DCs, BAFFR-Fc, CD4+ peptide, ICAs, IL-2 therapies, SAgAs, sCD73, RAD51 inhibitor, TLR9 inhibitor, and VD decreases diabetes development (**Figure 1A**). However, the role of CD73 knockdown (such as siRNA) and knockout in diabetes is unclear.

Many studies have shown that MSC transplantation, such as adipose tissue-derived MSCs, bone MSCs (BMSCs), human gingiva MSCs (GMSCs), pancreatic MSCs, human islet-MSCs (HI-MSCs), human umbilical cord MSCs (HUMSCs), Islet MSCs, pigment epithelial-derived factor (PEDF) gene-modified HUMSCs (PEDF-HUMSCs), umbilical cord (UC) MSCs (UC-MSCs), Wharton’s jelly-derived MSCs (WJMSCs) suppressed diabetes development (13, 16, 17, 22, 24–36). Human urine-derived stem cells (hUSCs) and their exosomes (hUSC-Exos) also suppressed diabetes development (37–39). Cow-derived placental stem cells (CPSCs) and periosteum-derived progenitor cells (PDPCs) promoted insulin secretion by differentiating into islet-producing cells (IPCs) (40, 41), suggesting that CPSCs and PDPCs have the potential to treat diabetes by differentiating IPCs. CD73 is expressed in hUSCs, hUSC-Exos, CPSCs, and PDPCs. As mentioned earlier, CD73 is a marker of MSCs. However, the role and mechanism of CD73 in MSCs, hUSCs, and USC-Exos in suppressing diabetes is unclear. Notably, MSCs, hUSCs, and USC-Exos can hydrolyze inflammatory extracellular ATP to anti-inflammatory adenosine via expressing CD73 and CD39 (42). In addition, GMSCs require CD39/CD73 signals to inhibit T1DM (43). GMSCs delayed the onset of diabetes by downregulating IL-17 and IFN-γ levels in CD4+ and CD8+ T cells in spleens, pancreatic lymph nodes (pLN), and other lymph nodes via expressing CD39 and CD73 (43). Thus, CD73 plays an anti-inflammatory role and immunoregulatory function in MSCs for treating diseases.

### 2.2 Atherosclerosis

Serum CD73 activity is upregulated in patients with atherosclerotic burden (44). Deficiency CD73 in patients exhibited extensive medial arterial calcification (MAC) which is an atherosclerosis risk, suggesting that CD73 may be a biomarker of MAC and atherosclerosis (45–47). Many studies have shown that CD37 is an enemy of atherosclerosis. For example, deficiency CD73 caused arterial calcification in patients, suggesting that overexpression CD73 suppressed arterial calcification (45–47). In ECs, CD73 suppressed inflammation and thrombosis and enhanced endothelial permeability by activating the adenosine/P1 receptor signaling pathway, (48). ECs CD73 can reduce leukocyte adhesion to the endothelium. Deletion of ECs connexin40 (Cx40) increases atherosclerosis by increasing CD73-dependent leukocyte adhesion via reducing CD73 expression (49), suggesting that ECs CD73 is an antiatherosclerotic factor. Aspirin reduces atherosclerotic plaque and immuno-inflammation by rebalancing Treg/Th17 cells via enhancing CD73 expression in ApoE^-/-^ mice (50). However, CD37 is a friend of atherosclerosis. Suppressing CD73 with siRNA decreased atherosclerosis and plaque formation by reducing migration, proliferation, and foam cell transformation of vascular smooth muscle cells (VSMCs) via reducing CyclinD1 expression and serum lipid levels in ApoE-/- mice (51). However, the mechanism of CD73 on serum lipid levels is unclear. Inhibition of CD73 can reduce the increase of heart rate caused by hypoxia (52). Benzothiadiazine derivatives, the CD73 inhibitors, were investigated for treating atherosclerosis and ischemia-reperfusion injury (53). These results suggest that CD73 is double-sided in atherosclerosis. ECs CD73 is an antiatherosclerotic factor, while VSMCs CD73 is a proatherosclerotic factor. In fact, whether CD73 is a friend or foe in atherosclerosis may be age-related (**Figure 1B**). In early atherosclerosis, CD73 knockout promoted plaque area in apoE^-/-^ mice at 12 weeks of age. However, the pattern shifts with age. CD73 knockout did not plaque area in apoE^-/-^ mice at 20 weeks of age. In apoE^-/-^ mice at 32 weeks and 52 weeks of age, CD73 knockout decreased plaque area by reducing lipolysis (54). ADO suppresses lipolysis, suggesting that CD73 promoted plaque accumulation by suppressing lipid catabolism via catalyzing the conversion of AMP to ADO with aging (54). Thus, with the increase of age, CD73 gradually changed from inhibiting atherosclerosis to promoting atherosclerosis.

## 3 The agent development in preclinical and clinical trials by targeting CD73

### 3.1 Targeting CD73

Given the important role of CD73 in diabetes mellitus and atherosclerosis, we searched for agents that target CD73 with AdisInsight, Bing, Chinadrugtrials, ClinicalTrials, Glgoo, ICTRP, Pharmacodia, Pharnexcloud, Pubmed, Yaozh, and Zhihuiya. Indeed, many agnets were developed in preclinical and clinical trials by targeting CD73 (**Table 1**), including A000830 (also named A-001190, A-001202, A-001421, AB-421) (55, 56), Adeno-associated viral type 5 (AAV5)-CD39/CD73 (57), ABSK051 (58–60), AG-2170 (61), AK131 (also named AK123) (62), ATN-037 (also named ATG-037, CB-708) (63–65), AP401 (67–69), APB-A2 (also named Anti-CD73 IgG4) (66), APCP (70), BB-1709 (71), BC010 (72), BP-1200 (73, 74), BPI-472372 (75, 76), BR101 (also named Ansipastobart) (77), BsAb CD73xEGFR (78), BMS-986179 (79), CBO421 (also named CBO-212, CD-421) (80–83), CC-5 (84), CD39/CD73 bifunctional fusion protein (85), CD39/CD73 transgenic exosomes and recombinant fusion protein (86, 87), CD73/PD-1 targeting DFC (88, 89), CD73 inhibitor (BioArdis) (90), CD73 inhibitor (Arcus Bios) (91), CD73 ASO (92), CHS-7304 (93), Compound 12f (94), Dalutrafusp alfa (also named AEGN-1423, GS-1423) (95), DN-018 (also named DN-019, DN-020, DN-052, DN-A1) (96), Dresbuxelimab (also named ak-119) (97), FP-1201 (also named ATC code L03AB07, Avonex, BG9418, Rebif, FP-1201-lyo, MR11A8, Traumakine) (98–101), ^68^GA-DOTA-dPNE, GB-7002 (also named GB-7002-01, GB-7002-04) (102), GI-108 (103, 104), HB-0039 (105), HB0045 (106), HB0046 (107), HB-0052 (106), HBM1007 (108), HLX23 (109), IBI325 (110), INCA-00186 (also named INCA-0186) (111), IOA-237 (112), IPH5301 (113 **),** JAB-X1800 (also named CD73-STING iADC) (114, 115), JAB-BX102 (116), LY-3475070 (84), mAb19 (117), Mupadolimab (also named CPI-006, CPX-006) (118–121), Oleclumab (also named MEDI9447) (122–124), OP-5558 (125), OP-5244 (126, 152), OPN-CD73 (also named OPN-9627) (127), ORIC-533 (also named OP-5244, OR-558) (128–130), PBF2828 (131), PM-1015 (132), PSB-12379 (133), PSB-18332 (134, 135), PSB-19416 (134, 135), PT199 (136), Quemliclustat (also named AB680, A-0002396) (137–139, 152–155), S095024 (also named Sym024) (140), SHR170008 (141), siRNA-CD73 (70), SRF-373 (also named NZV930) (142), TRB-010 (143), Uliledlimab (also named TJD5, TJ004309, I-Mab Biopharma) (144–146), VE-3771 (147), VE-5953 (147), X-6350 (148), ZM514 (149), ZM552 (150), ZM553 (150), ZM557 (150), and ZS-1001 (151). However, no agents have been approved for sale. Notably, most of these agents are used to treat cancer. Whether CD73 as a target for treating atherosclerosis and diabetes is worth developing remains to be investigated. Notably, CD73 may be a delivery target for atherosclerotic plaques. As mentioned earlier, CD73 is a specific marker of MSC. Many studies have shown that umbilical cord (UC) MSC transplantation suppressed atherosclerosis development. However, MSCs have limited homing ability to atherosclerotic plaque sites. Integrin beta 3 (ITGB3)-modified MSCs successfully retained CD73 expression and enhanced the plaque-homing ability of MSCs, suggesting that ITGB3 is a good material for MSCs to deliver to plaques (7). CD73 combination with ITGB3 may be worth developing as the delivery target for atherosclerotic plaques.

**Table 1:** Agents that target CD73 in preclinical and clinical trials.

Name	Target	Introduce/Administration	Diseases	Status/Date	Developer	Refs
A000830	CD73 inhibitor	Small molecule inhibitor, SC	Cancer, such as CRC	Preclinical trials	Arcus Biosciences, Inc.	(55, 56)
AAV5-CD39/CD73	CD39 agonist, CD73 agonist	AAV5 vector-mediated expression of CD39 and CD73	Inflammatory disease, such as APSI	Preclinical trials	Arthrogen B. V; Academic Medical Center/University of Amsterdam	(57)
ABSK051	CD73 antagonist	Small molecule inhibitor. ABSK051 (IV) +- Tislelizumab	Solid tumors	Phase 1 (Ongoing on 03 January 2024)	Abbisko	CTR20233817, (58–60)
AG-2170	CD73 inhibitor	Unknown	Oncology	Preclinical trials	Angarus Therapeutics, Inc	(61)
AK131	anti-PD-1 and CD73 BsAb	An humanized IgG1 subtype BsAb that targeting CD73 and PD-1, AK131 alone (IV)	Advanced solid tumors	Phase 1 (Recruiting on 17 January 2024)	Akeso, Inc	NCT06166888, (62)
ATN-037	CD73 inhibitor	Small molecule inhibitor. ATN-037 (Oral) plus Keytruda (also named Pembrolizumab, MK-3475)	Locally advanced or metastatic solid tumors	Phase 1/1b (Recruiting on 30 April 2024)	Antengene Therapeutics Limited, Merck Sharp & Dohme LLC	NCT05205109, (63–65)
APB-A2	CD73 inhibitor	A humanized anti-CD73 mAb	Solid tumors	Phase 1 (Discontinued on 31 March 2023)	Aprilbio Co. Ltd	(66)
AP401	CD155 inhibitor, CD73 inhibitor	An iPSC Bi-specific CAR NK cell targeting CD155 and CD73	Solid tumors	IND (Plan on 2024)	Alphageneron Pharmaceuticals Inc	(67–69)
APCP	CD73 inhibitor	Small molecule inhibitor	Pancreatic cancer	Preclinical trials	The University of Texas Health Science Center at Houston	(70)
BB-1709	ADC CD73 inhibitor	IV	Locally advanced/​metastatic solid tumors	Phase 1 (Recruiting on 05 February 2024)	Bliss Biopharmaceutical (Hangzhou) Co., Ltd	NCT06241898, CXSL2200580, (71)
BC010	CD73 inhibitor	A humanized anti-CD73 mAb	Solid tumor	Preclinical trials	Dragonboat Biopharmaceutical	(72)
BP-1200	CD73 inhibitor	A humanized anti-CD73 mAb	Cancer	Preclinical trials	BrightPath Biotherapeutics	(73, 74)
BPI-472372	CD73 inhibitor	Small molecule inhibitor. BPI-472372 (Oral)	Advanced solid tumors	Phase 1a (Recruiting on 21 August 2023)	Betta Pharmaceuticals	(75, 76)
BR101	CD73 inhibitor	A fully humanized anti-CD73 mAb, BR101 (IV)	Advanced solid tumors	Phase 1a (Recruiting on 21 August 2023)	BioRay Pharmaceutical Co., Ltd	NCT06001580, CXSL2101026, ChiCTR2100049016, (77)
BsAb CD73xEGFR	CD73 inhibitor, EGFR inhibitor	A humanized anti-CD73/EFGR BsAb	Cancer	Preclinical trials	University of Groningen, University Medical Center Groningen	(78)
BMS-986179	CD73 inhibitor	A humanized anti-CD73 mAb, BMS-986179 alone (IV) +- Nivolumab or +- rHuPH20	Solid cancers	Phase 1/2a (Completed on 05 April 2023)	Bristol Myers Squibb Co	NCT02754141, (79)
CBO421	CD73 inhibitor	A first-in-class DFC that targets CD73. 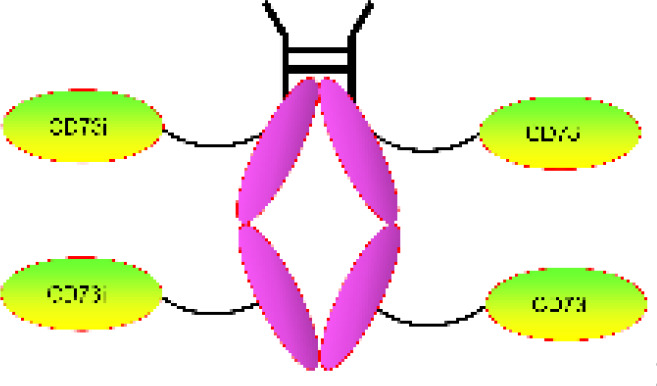 , This information was modified from Ref (80)	Solid tumors	IND application (Approved by FDA on 31 July 2024)	Cidara Therapeutics, Inc	(80–83)
CC-5	CD73 inhibitor PD-L1 inhibitor	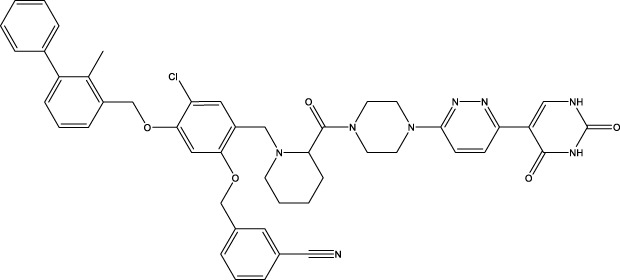	Cancer	Preclinical trials	Wenzhou Medical University	(84)
CD39/CD73 BFP	CD39 agonist CD73 agonist	BFP	Inflammatory diseases	Preclinical trials	Beth Israel Deaconess Medical Center and Harvard Medical School	(85)
CD39/CD73 transgenic exosomes and recombinant fusion protein	CD39 agonist CD73 agonist	A soluble CD39/CD73 transgenic exosomes and RAIN fusion protein	Inflammatory disease, such as APSI	Preclinical trials	Arthrogen B. V; Academic Medical Center/University of Amsterdam	(86, 87)
CD73/PD-1 targeting DFC	CD73 inhibitor, PD-1 inhibitor	Dual-acting CD73/PD-1 targeting DFC candidate	Solid tumors	Preclinical trials	Cidara Therapeutics Inc	(88, 89)
CD73 inhibitor (BioArdis)	CD73 inhibitor	Small molecule inhibitor	Cancer	Preclinical trials	BioArdis LLC	(90)
CD73 inhibitor (Arcus Bios)	CD73 inhibitor	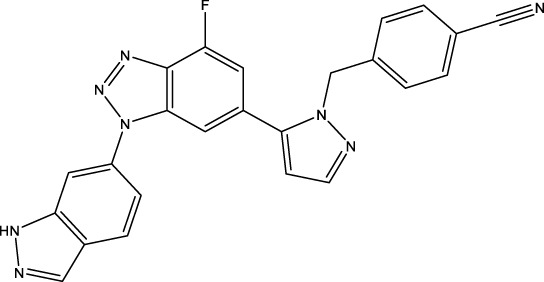 CD73: IC50, 12 nM	Cancer	Preclinical trials	Arcus Biosciences, Inc	(91)
CD73 inhibitor (Arcus Bios)	CD73 inhibitor	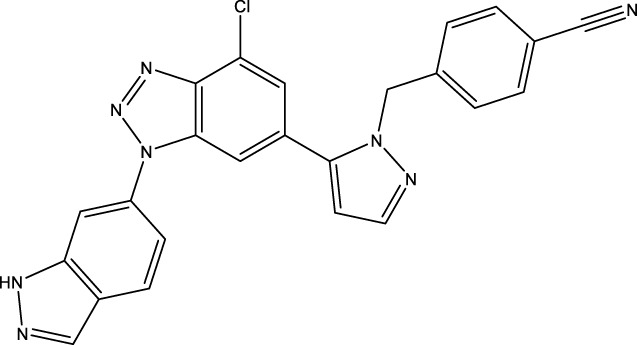 CD73: IC50, 19 nM	Cancer	Preclinical trials	Arcus Biosciences, Inc	(91)
CD73 ASO	CD73 inhibitor	Antisense oligonucleotides	Cancer	Preclinical trials	Secarna Pharmaceuticals GmbH & Co. KG	(92)
CHS-7304	CD73 inhibitor	Small molecule inhibitor	Solid tumors	Preclinical trials	Coherus BioSciences, Inc	(93
Compound 12f	CD73 inhibitor	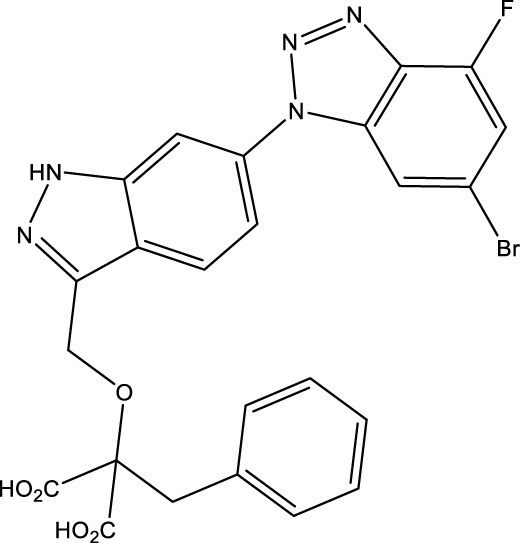 CD73, IC50, 60 nM	Cancer	Preclinical trials	East China University of Science and Technology	(94)
Dalutrafusp alfa	CD73 inhibitor, TGFβ inhibitor	An anti-CD73-TGFβ-Trap Bifunctional Antibody. Dalutrafusp alfa (IV) +- mFOLFOX6	Advanced solid tumors	Phase 1 (Discontributed on 21 November 2023)	Gilead Sciences	NCT03954704, (95)
Dalutrafusp alfa	CD73 inhibitor, TGFβ inhibitor	Dalutrafusp alfa (IV) + Botensilimab +- Chemotherapy (gemcitabine and nab-paclitaxel)	Advanced pancreatic cancer	Phase 2 (Recruiting on 13 August 2024)	Bruno Bockorny; Agenus Inc; Dana-Farber Cancer Institute	NCT05632328
Dalutrafusp alfa	CD73 inhibitor, TGFβ inhibitor	Dalutrafusp alfa (IV) + Botensilimab + Balstilimab	CRC liver metastases	Phase 2 (Recruiting on 12 April 2024)	Agenus Inc; Weill Medical College of Cornell University	NCT06300463
DN-018	CD73 inhibitor, TLR8 agonist	Small molecule inhibitor	Cancer	Preclinical trials	Shanghai *De Novo* Pharmatech	(96)
Dresbuxelimab	CD73 inhibitor	A humanized anti-CD73 mAb. Dresbuxelimab (IV)	Advanced or metastatic solid tumors	Phase 1 (Completed on 25 April 2024)	Akeso Biopharma, Tianjin Medical University Cancer Insitute & Hospital, Shanghai Zhongshan Hospital	NCT05173792
Dresbuxelimab	CD73 inhibitor	Dresbuxelimab (IV) plus AK104 (an anti-PD-1 and CTLA-4 bispecific antibody)	Advanced solid tumors	Phase 1b/2 (Recruiting on 30 December 2022)	Akeso Biopharma, Tianjin Medical University Cancer Insitute & Hospital	NCT05559541
Dresbuxelimab	CD73 inhibitor	Dresbuxelimab (IV) + AK104	Advanced or metastatic solid tumors	Phase 1 (Active, not recruiting on 06 January 2023)	Akeso Biopharma	NCT04572152
Dresbuxelimab	CD73 inhibitor	Dresbuxelimab (IV) + AK112 (an anti-VEGF and PD-1 bispecific antibody)	Advanced solid tumors	Phase 1b//2 (Recruiting on 20 April 2023)	Akeso Biopharma; Peking Union Medical College Hospital	NCT05689853
Dresbuxelimab	CD73 inhibitor	Dresbuxelimab alone (IV)	COVID-19	Phase 1 (Completed on 20 April 2023)	Akesobio Australia Pty Ltd	NCT04516564, (97)
Dresbuxelimab	CD73 inhibitor	Dresbuxelimab (IV) + AK112 +- Pemetrexed + Carboplatin	NSCLC	Phase 1//2 (Recruiting on 09 March 2023)	Akeso Biopharma; Guangdong Provincial People’s Hospital	NCT05636267
Dresbuxelimab	CD73 inhibitor	Dresbuxelimab (IV) + AK112 +- mFOLFOX6 or +- FOLFIRI	Advanced pMMR/MSS CRC	Phase 1b//2 (Not yet recruiting on 06 May 2023)	Akeso Biopharma, Cancer Hospital Affiliated to Harbin Medical University	NCT05846867
FP-1201	CD73 agonist, IFNβ-1a agonist	A recombinant human IFN β-1a. FP-1201 (IV)	Prevent toxicities after CD19-directed CAR T-Cell therapy	Phase 1/2 (Discontinued on 12 July 2024)	Faron Pharmaceuticals Ltd	NCT05936229
FP-1201	CD73 agonist, IFNβ-1a agonist	FP-1201 (IV)	Prevention of multi-organ failure in patients after open surgery for RAAA	Phase 2 (Discontinued on 14 January 2021)	Faron Pharmaceuticals Ltd	NCT03119701, (98)
FP-1201	CD73 agonist, IFNβ-1a agonist	FP-1201 (IV)	ALI and ARDS	Phase 1/2 (Completed on 27 May 2015)	Faron Pharmaceuticals Ltd	NCT00789685, (99, 100)
FP-1201	CD73 agonist, IFNβ-1a agonist	FP-1201 (IV)	ARDS	Phase 3 (Discontinued due to inefficacy on 30 March 2020)	Faron Pharmaceuticals Ltd	NCT02622724, (101)
FP-1201	CD73 agonist, IFNβ-1a agonist	FP-1201 (IV) VS Dexamethasone	COVID-19	Phase 2 (Discontinued due to the decrease of the epidemic on 21 July 2023)	Faron Pharmaceuticals Ltd	NCT04860518
^68^GA-DOTA-dPNE	CD73 inhibitor	A ^68^GA-labeled CD73 targeting probe	Breast cancer (Diagnosis)	Phase 1 (Not yet recruiting on 10 May 2024)	Peking Union Medical College Hospital	ChiCTR2400083919
GB-7002	CD73 inhibitor	A humanized anti-CD73 mAb	GC	Preclinical trials	Biotheus; Shanghai Genechem	(102)
GI-108	CD73 inhibitor, IL-2RA inhibitor	A BFP that targets CD73 and IL-2RA	Cancer	Preclinical trials	GI Innovation, Inc	(103)
GI-108			Cancer	IND application (Plan on 2024)	GI Innovation, Inc	(104)
HB-0039	CD73 inhibitor	Drug conjugates	Solid tumors	Preclinical trials (Discontinued on 07 November 2023)	Huabo Biopharm (Shanghai) Co., Ltd	(105)
HB0045	CD73 inhibitor	A humanized anti-CD73 mAb. HB0045 alone (IV)	Advanced solid tumors	Phase 1 (Recruiting on 23 October 2023)	Shanghai Huaota Biopharmaceutical Co., Ltd; Gabrail Cancer Center Research; Dana-Farber Cancer Institute; M.D. Anderson Cancer Center	NCT06056323, (106)
HB0046	CD39 inhibitor, CD73 inhibitor	A humanized anti-CD39/​CD73 BsAb	Cancer	Phase 1 (Approved on 10 July 2024)	Shanghai Huaota Biopharmaceutical Co., Ltd	(107)
HB-0052	CD73 inhibitor	ADC	Advanced solid tumors	Phase 1/2 (Recruiting on 04 September 2024)	Shanghai Huaota Biopharmaceutical Co., Ltd	CTR20242618, (106)
HBM1007	CD73 inhibitor	A humanized Anti-CD73 mAb	Solid tumors	Phase 1 (Approved on 19 January 2023)	Harbor BioMed	(108)
HLX23	CD73 inhibitor	A fully humanized recombinant anti-CD73 mAb. HLX23 (IV)	Advanced or metastatic solid tumors	Phase 1 (Discontinued due to reevaluating the study design on 17 January 2023)	Shanghai Henlius Biotech	NCT04797468, (109)
IBI325	CD73 inhibitor	A fully humanized anti-CD73 mAb. IBI325 (IV) +- sintilimab	Advanced solid tumors	Phase 1 (Completed on 18 August 2023)	Innovent Biologics (Suzhou) Co. Ltd	NCT05119998, (110)
IBI325	CD73 inhibitor	A humanized anti-CD73 mAb. IBI325 (IV) +- sintilimab	Advanced solid tumors	Phase 1 (Unknown status on 18 February 2022)	Shandong Cancer Hospital and Institute	NCT05246995
IBI325	CD73 inhibitor	IBI325 (IV) or +- IBI363	Advanced solid tumors	Phase 1/2 (Recruiting on 30 May 20240)	Hunan Province Tumor Hospital, Xiangya Hospital of Central South University	NCT06081907
INCA-00186	CD73 inhibitor	A humanized anti-CD73 mAb. INCA-00186 (IV) +- retifanlimab +- INCB106385	Advanced solid tumors	Phase 1 (Active, not recruiting on 06 June 2024)	Incyte Corporation	NCT04989387
INCA-00186	CD73 inhibitor		Gastrointestinal cancer; solid tumors; SCC	Phase 1 (Discontinued on 02 May 2023)	Incyte Corporation	(111)
IOA-237	CD73 inhibitor	A humanized anti-CD73 mAb	Solid tumors	Preclinical trials	iOnctura SA	(112)
IPH5301	CD73 inhibitor	A humanized anti-CD73 mAb. IPH5301 (IV) +- paclitaxel and trastuzumab	Advanced solid tumors	Phase 1 (Recruiting on 20 March 2024)	Institut Paoli-Calmettes	NCT05143970, (113)
JAB-BX100	CD73 inhibitor, STING inhibitor	A humanized anti-CD73 mAb	Cancer	Phase 2 (Plan on 26 July 2023)	Jacobio Pharmaceuticals Group Co., Ltd	(114)
JAB-X1800	CD73 inhibitor, STING agonist	A conjugating potent STING agonist to CD73 mAb 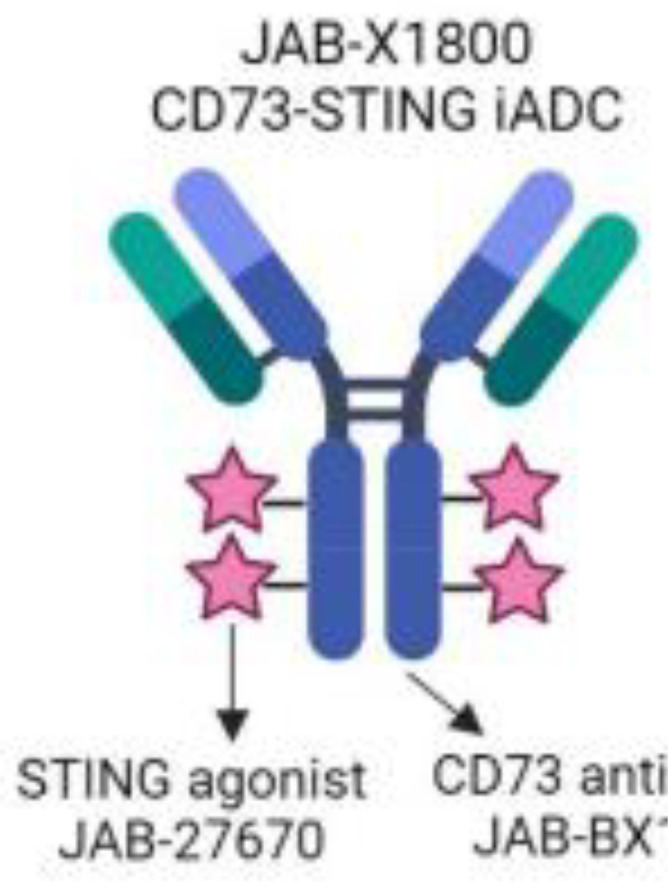 This information was modified from Ref (115)	Cancer	Preclinical trials	Jacobio Pharmaceuticals Group Co., Ltd	(115)
JAB-BX102	CD73 inhibitor	A humanized anti-CD73 mAb. JAB-BX102 (IV) +- pembrolizumab (anti-PD-1 mAb)	Advanced solid tumors	Phase 1/2a (Recruiting on 22 March 2024)	Jacobio Pharmaceuticals Co., Ltd.	NCT05174585, (116)
LY-3475070	CD73 inhibitor	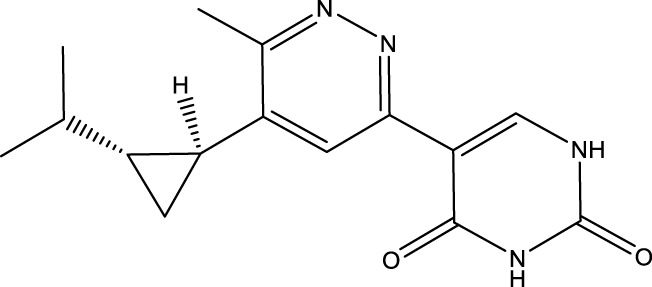 PubChem CID: 152262911. LY-3475070 (Oral) +- pembrolizumab	Advanced cancer	Phase 1 (Completed on 05 April 2024)	Eli Lilly & Co; Merck Sharp & Dohme LLC	NCT04148937, (84)
mAb19	CD73 inhibitor	A humanized anti-CD73 mAb	Solid tumors	Preclinical trials	Boehringer Ingelheim GmbH	(117)
Mupadolimab	Anti-CD73 mAb	A humanized anti-CD73 mAb. Mupadolimab (IV) +- ciforadenant or pembrolizumab	Advanced cancer	Phase 1 (Completed on 21 December 2023)	Corvus Pharmaceuticals, Inc.	NCT03454451, (118–120)
Mupadolimab	Anti-CD73 mAb	Mupadolimab (IV) +- SOC	COVID-19	Phase 1 (Completed on 21 July 2021)	Corvus Pharmaceuticals, Inc.	NCT04464395, (121)
Mupadolimab	Anti-CD73 mAb	Mupadolimab (IV) +- SOC	COVID-19	Phase 3 (Discontinued on 21 September 2022)	Corvus Pharmaceuticals, Inc.	NCT04734873
Oleclumab	Anti-CD73 mAb	Anti-CD73 mAb. Oleclumab (IV)	Advanced solid malignancies	Phase 1 (Completed on 08 July 2019)	AstraZeneca	NCT03736473
Oleclumab	Anti-CD73 mAb	Oleclumab (IV) + MEDI4736 (also named Durvalumab, IMFINZI, PD-L1 inhibitor)	Advanced solid tumors	Phase 1 (Completed on 11 July 2023)	MedImmune LLC	NCT02503774, (122, 123)
Oleclumab	Anti-CD73 mAb	Oleclumab (IV) +- MEDI4736	MIBC	Phase 1 (Completed on 13 September 2022)	AstraZeneca; Dana-Farber Cancer Institute	NCT03773666
Oleclumab	Anti-CD73 mAb	Oleclumab (IV) + Paclitaxel + Carboplatin + Durvalumab	Locally recurrent inoperable or metastatic TNBC	Phase 1/2 (Active, not recruiting on 21 September 2023)	AstraZeneca; Jules Bordet Institute	NCT03616886
Oleclumab	Anti-CD73 mAb	Oleclumab (IV) + osimertinib or AZD4635	mPDAC	Phase 1b/2 (Active, not recruiting on 20 August 2024)	MedImmune LLC	NCT03381274
Oleclumab	Anti-CD73 mAb	Oleclumab (IV) +- MEDI4736	Relapsed OC	Phase 2 (Completed on 11 September 2023)	Nordic Society of Gynaecological Oncology - Clinical Trials Unit; GCIG; ENGOT	NCT03267589
Oleclumab	Anti-CD73 mAb	Oleclumab (IV) + MEDI4736	Multi-cancer	Phase 2 (Discontinued on 17 November 2020)	AstraZeneca; University Health Network (Toronto); Princess Margaret Cancer Centre	NCT04262375
Oleclumab	Anti-CD73 mAb	Oleclumab (IV) + MEDI4736	Advanced NSCLC with PD-1 inhibitor resistance	Phase 2 (Active, not recruiting on 03 May 2024)	Assistance Publique Hopitaux De Marseille	NCT03833440
Oleclumab	Anti-CD73 mAb	Oleclumab (IV) + MEDI4736	Resectable PDAC	Phase 2 (Recruiting on 08 January 2024)	University Health Network (Toronto)	NCT06060405
Oleclumab	Anti-CD73 mAb	Oleclumab (IV) + MEDI4736	HNSCC, PDAC, NSCLC	Phase 2 (Discontinued due to inefficacy on 17 November 2020)	University Health Network (Toronto); Princess Margaret Cancer Centre	NCT04262388
Oleclumab	Anti-CD73 mAb	Oleclumab (IV) + MEDI4736	Stage III unresectable NSCLC	Phase 3 (Recruiting on 28 August 2024)	AstraZeneca; Gustave Roussy, Cancer Campus, Grand Paris	NCT05221840
Oleclumab	Anti-CD73 mAb	Oleclumab (IV) + AZD4635	PSa	Phase 2 (Completed on 16 April 2024)	AstraZeneca	NCT04089553
Oleclumab	Anti-CD73 mAb	Oleclumab (IV) + MEDI4736	Recurrent, refractory, or metastatic sarcoma	Phase 2 (Recruiting on 03 July 2024)	M.D. Anderson Cancer Center; National Cancer Institute (NCI)	NCT04668300
Oleclumab	Anti-CD73 mAb	Oleclumab (IV) + MEDI4736 + Stereotactic Body Radiotherapy	Luminal B BC	Phase 2 (Active, not recruiting on 17 June 2024)	Jules Bordet Institute; AstraZeneca	NCT03875573
Oleclumab	Anti-CD73 mAb	Oleclumab (IV) + MEDI4736 + nab-paclitaxel + gemcitabine	Resectable/​borderline resectable primary PC	Phase 2 (Recruiting on 06 May 2024)	M.D. Anderson Cancer Center	NCT04940286
Oleclumab	Anti-CD73 mAb	Oleclumab (IV) + MEDI4736	Resectable NSCLC	Phase 2 (Completed on 24 February 2022)	MedImmune LLC	NCT03794544
Oleclumab	Anti-CD73 mAb	Oleclumab (IV) + MEDI4736 +- chemotherapy	NSCLC	Phase 1b (Active, not recruiting on 16 July 2024)	AstraZeneca; UCSD Morres Cancer Center; National Taiwan University Hospital	NCT03819465
Oleclumab	Anti-CD73 mAb	Oleclumab (IV) + MEDI4736 + mFOLFOX6	MSS-CRC	Phase 2 (Discontinued due to changes in the standard of care on 21 September 2020)	MedImmune LLC	NCT04145193
Oleclumab	Anti-CD73 mAb	Oleclumab (IV) + FOLFOX + Bevacizumab + Durvalumab	MSS-CRC	Phase 1b/2 (Discontinued due to no superior efficacy on 17 February 2022)	MedImmune LLC	NCT04068610
Oleclumab	Anti-CD73 mAb	Oleclumab (IV) + MEDI4736 + paclitaxel	Metastatic TNBC	Phase 1/2 (Active, not recruiting on 14 August 2024)	AstraZeneca, Barts Cancer Institute	NCT03742102
Oleclumab	Anti-CD73 mAb	Oleclumab (IV) + MEDI4736 + standard RT	NSCLC	Phase 1 (Recruiting on 31 July 2024)	National Cancer Institute (NCI); NRG Oncology	NCT03801902
Oleclumab	Anti-CD73 mAb	Oleclumab (IV) + MEDI4736 + Platinum doublet chemotherapy (CTX)	NSCLC	Phase 2 (Recruiting on 28 August 2024)	AstraZeneca; Parexel; MD Anderson Cancer Center Houston	NCT05061550
Oleclumab	Anti-CD73 mAb	Oleclumab (IV) + MEDI4736 + AZD4635	NSCLC	Phase 2 (Active, not recruiting on 12 June 2024)	AstraZeneca; The University of Texas MD Anderson Cancer Center	NCT03334617
Oleclumab	Anti-CD73 mAb	Oleclumab (IV) + MEDI4736	NSCLC	Phase 2 (Completed on 12 December 2023)	MedImmune LLC	NCT03822351, (124)
Oleclumab	Anti-CD73 mAb	Oleclumab (IV) + MEDI4736	Advanced solid malignancies	Phase 2 (Completed on 26 May 2023)	AstraZeneca; SCRI Development Innovations, LLC	NCT02740985
OP-5558	CD73 inhibitor	Small molecule inhibitor that is an analog of ORIC-533	MM	Preclinical trials	Oric Pharmaceuticals, Inc	(125)
OP-5244	CD73 inhibitor	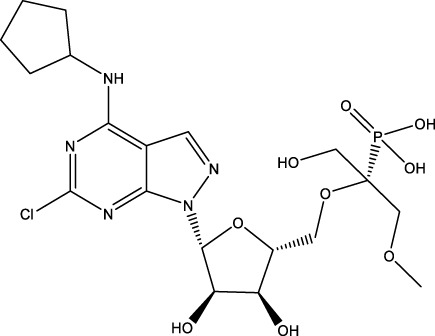 Oral	Cancer, such as PC	Preclinical trials	Oric Pharmaceuticals, Inc	(126)
OPN-CD73	CD73 inhibitor	Small molecule inhibitor, oral	Cancer	Preclinical trials	Opna Bio SA	(127)
ORIC-533	CD73 inhibitor	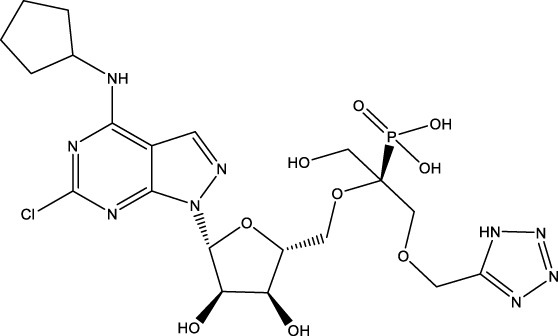 Oral	Relapsed or refractory MM	Phase 1b (May comlpete on 2024)	Oric Pharmaceuticals, Inc	NCT05227144, (128, 129)
ORIC-533			Solid tumors	Preclinical trials (Discontinued on 11 May 2024)	Oric Pharmaceuticals, Inc	(130)
PBF2828	CD39 inhibitor, CD73 inhibitor	A humanized anti-CD39/​CD73 BsAb	Cancer	Preclinical trials (Discontinued on 14 May 2019)	Palobiofarma SL	(131)
PM-1015	CD73 inhibitor	A humanized anti-CD73 mAb	Advanced solid tumors	Phase 1 (Recruiting on 18 July 2023)	Biotheus Inc	NCT05950815, (132)
PSB-12379	CD73 inhibitor	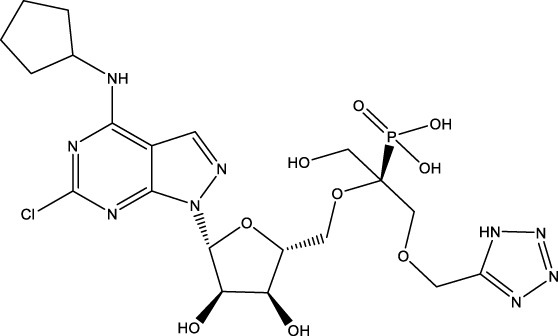	Cancer	Preclinical trials	University of Bonn	(133)
PSB-18332	CD73 inhibitor	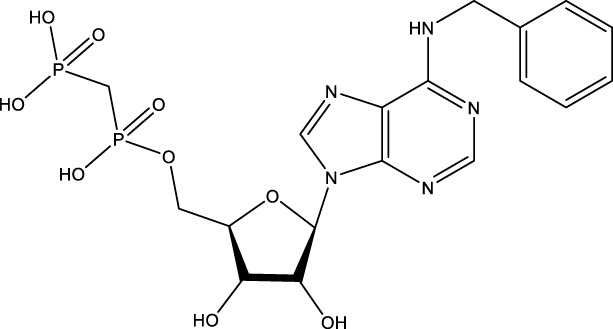	Cancer	Preclinical trials	University of Bonn	(134, 135)
PSB-19416	CD73 inhibitor	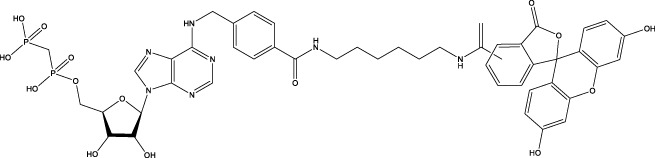	Cancer	Preclinical trials	University of Bonn	(134, 135)
PT199	CD73 inhibitor	A humanized anti-CD73 mAb. PT199 (IV) +- tislelizumab (PD-1 inhibitor)	Advanced solid tumors	Phase 1 (Recruiting on 13 June 2024)	Phanes Therapeutics, Inc	NCT05431270, (136)
Quemliclustat	CD73 inhibitor	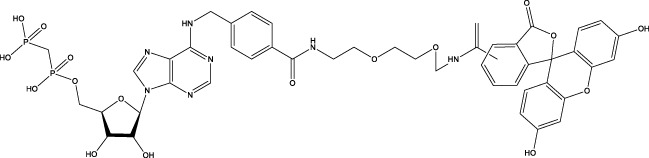 Quemliclustat (Oral)	Healthy volunteers	Phase 1 (Completed on 2024-05-24)	Arcus Biosciences, Inc	NCT04575311
Quemliclustat	CD73 inhibitor	Quemliclustat (IV)	Healthy volunteers	Phase 1 (Completed on 2024-05-24)	Arcus Biosciences, Inc	NCT03677973, (137)
Quemliclustat	CD73 inhibitor	Quemliclustat (IV) +-Zimberelimab (also named AB122, a fully human anti-PD-1 mAb) + nab-paclitaxel(NP) & gemcitabine (Gem)	Advanced PCa	Phase 1 (Recruiting on 2024-09-19)	Arcus Biosciences, Inc	NCT04104672
Quemliclustat	CD73 inhibitor	Quemliclustat (IV) + zimberelimab + + gemcitabine + cisplatin	Advanced BTCs	Phase 2 (Recruiting on 2024-09-19)	Big Ten Cancer Research Consortium; Arcus Biosciences, Inc; Gilead Sciences; University of Wisconsin	NCT06048133
Quemliclustat	CD73 inhibitor	Quemliclustat (IV) + Etrumadent (also named AB928, a dual A2aR/A2bR antagonist) + Zimberelimab	Metastatic CRC	Phase 1b/2 (Active, not recruiting on 2024-09-20)	Arcus Biosciences, Inc; Gilead Sciences	NCT04660812
Quemliclustat	CD73 inhibitor	Quemliclustat (IV) + Stereotactic body radiotherapy (SBRT) + Zimberelimab + Etrumadenant	Localized PDAC	Phase 2 (Recruiting on 2024-02-28)	Columbia University; Arcus Biosciences, Inc	NCT06048484
Quemliclustat	CD73 inhibitor	Quemliclustat (IV) + Stereotactic body radiotherapy (SBRT) + Zimberelimab + Etrumadenant + Modified FOLFIRINOX	HSOPC	Phase 2 (Recruiting on 2024-05-07)	Columbia University; Arcus Biosciences, Inc	NCT05915442
Quemliclustat	CD73 inhibitor	Quemliclustat (IV) +- Zimberelimab + Etrumadenant	mCRPC	Phase 1b/2 (Completed on 2024-09-19)	Gilead Sciences; Arcus Biosciences, Inc	NCT04381832
Quemliclustat	CD73 inhibitor	Quemliclustat (IV) + Zimberelimab +- Docetaxel or +- Platinum Doublet Chemotherapy +-Domvanalimab	Advanced NSCLC	Phase 2 (Recruiting on 2024-09-20)	Gilead Sciences; Arcus Biosciences, Inc	NCT05676931
Quemliclustat	CD73 inhibitor	Quemliclustat (IV) +Zimberelimab	Advanced UGTM	Phase 2 (Recruiting on 2024-09-19)	Gilead Sciences; Arcus Biosciences, Inc	NCT05329766
Quemliclustat	CD73 inhibitor	Quemliclustat (IV) + Zimberelimab + Chemotherapy (Fluorouracil, Irinotecan, Leucovorin, Leucovorin Calcium, and Oxaliplatin)	Borderline resectable and locally advanced pancreatic adenocarcinoma	Phase 1/2 (Discontinued on 2024-02-01)	Jonsson Comprehensive Cancer Center; Arcus Biosciences, Inc	NCT05688215
Quemliclustat	CD73 inhibitor	Quemliclustat (IV) +- Chemotherapy	mPDAC	Phase 3 (Planned on 2024)	Arcus Biosciences, Inc	(138, 139)
Quemliclustat	CD73 inhibitor		PCa	Discontinued	Arcus Biosciences; Gilead Sciences; UCLAs Jonsson Comprehensive Cancer Center	(139)
S095024	CD73 inhibitor	A humanized anti-CD73 mAb. S095024 (IV) + cemiplimab	Advanced NSCLC	Phase 1b/2 (Recruiting on 14 August 2024)	Servier Bio-Innovation LLC	NCT06162572
S095024	CD73 inhibitor	S095024 (IV) +- Sym021 (Anti-PD-1)	Advanced solid tumor malignancies	Phase 1 (Active, not recruiting on 02 May 2024)	Symphogen A/S	NCT04672434, (140)
SHR170008	CD73 inhibitor	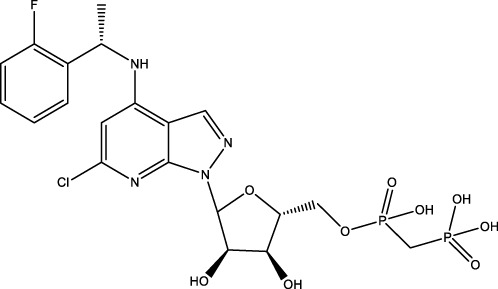	Cancer	Preclinical trials	Eternity Bioscience, Inc; Shanghai Hengrui Pharmaceutical Co. Ltd	(141)
siRNA-CD73	CD73 inhibitor	CD73 siRNA	Cancer	Preclinical trials	Universidade Federal de Ciências da Saúde de Porto Alegre (UFCSPA)	(70)
SRF-373	CD73 inhibitor	A humanized anti-CD73 mAb. SRF-373 (IV) + KAZ954	Advanced solid tumors	Phase 1 (Terminated due to Business reasons on 12 July 2024)	Surface Oncology, Inc (Acquired by Coherus BioSciences); Novartis Pharmaceuticals	NCT04237649, (142)
TRB-010	CD73 inhibitor	A humanized anti-CD73 mAb	Cancer	Preclinical trials	Trican Biotechnology	(143)
Uliledlimab	CD73 inhibitor	A differentiated humanized IgG1κ anti-CD73 mAb. Uliledlimab (IV) + atezolizumab (A humanized anti-PD-L1 mAb)	Advanced or metastatic cancer	Phase 1 (Unknown status on 01 April 2022)	Tracon Pharmaceuticals Inc	NCT03835949, (144)
Uliledlimab	CD73 inhibitor	Uliledlimab (IV) + atezolizumab	Advanced or metastatic cancer	Phase 1 (No longer available on 28 April 2023)	I-Mab Biopharma US Limited	NCT04869501
Uliledlimab	CD73 inhibitor	Uliledlimab (IV) + atezolizumab	OC and selected solid tumors	Phase 2 (Completed on 12 October 2023)	I-Mab Biopharma US Limited	NCT05001347
Uliledlimab	CD73 inhibitor	Uliledlimab (IV) +- Toripalimab (Humanized monoclonal antibody to PD-1)	Advanced solid tumor	Phase 1/2 (Active, not recruiting on 15 April 2024)	TJ Biopharma Co., Ltd; I-Mab Biopharma HongKong Limited	NCT04322006, (145, 146)
VE-3771	CD73 inhibitor	Small molecules inhibitor	Cancer	Preclinical trials	Verseon Corp	(147)
VE-5953	CD73 inhibitor	Small molecules inhibitor	Cancer	Preclinical trials	Verseon Corp	(147)
X-6350	A2AR inhibitor, A2BR inhibitor, CD73 inhibitor, CSF1 inhibitor	Cell-activate modulators (DNA encoded, cancer), small molecule inhibitors	Cancer	Preclinical trials	X-Chem Inc; Xios Therapeutics	(148)
ZM514	CD73 inhibitor	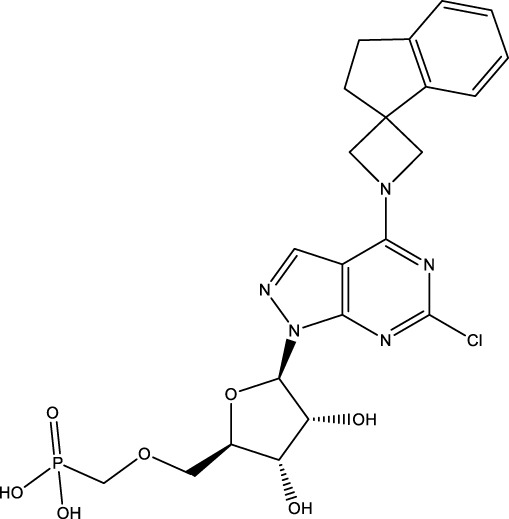	Cancer	Preclinical trials	The Second Military Medical University; The Second Military Medical University	(149)
ZM552	CD73 inhibitor	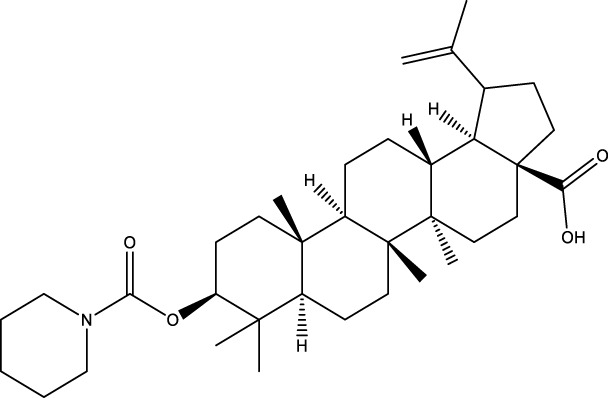	Cancer	Preclinical trials	The Second Military Medical University; Shanghai Institute of Technology	(150)
ZM553	CD73 inhibitor	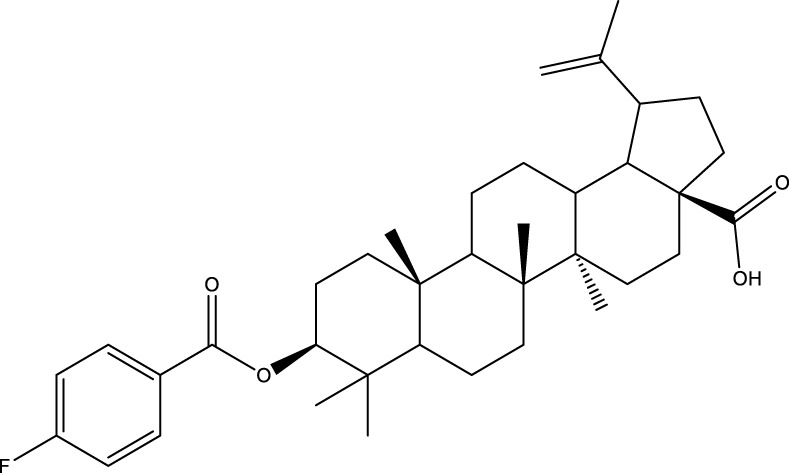	Cancer	Preclinical trials	The Second Military Medical University; Shanghai Institute of Technology	(150)
ZM557	CD73 inhibitor	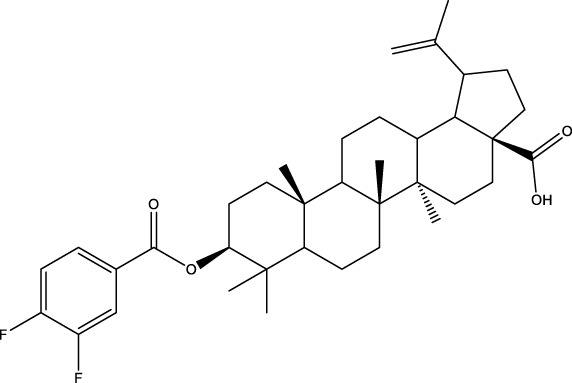	Cancer	Preclinical trials	The Second Military Medical University; Shanghai Institute of Technology	(150)
ZS-1001	CD73 inhibitor	Unknown	Solid tumor	Preclinical trials	Genuine Biotech Limited	(151)

The types and groups were obtained from AdisInsight, Bing, Chinadrugtrials, ClinicalTrials, Glgoo, ICTRP, Pharmacodia, Pharnexcloud, Pubmed, Yaozh, and Zhihuiya. AAV5, Adeno-associated viral type 5; ADCC, antibody-dependent cellular cytotoxicity; ALI, acute lung injury; APSI, air pouch synovial inflammation; ARDS, acute respiratory distress syndrome; ASO, antisense oligonucleotides; BC, Breast Cancer; BsAb, bispecific monoclonal antibody; BFP, bifunctional fusion protein; BTCs, biliary tract cancers; COVID-19, coronavirus disease 2019; CRC, colon cancer; DFC, drug Fc-conjugate; ENGOT, European Network of Gynaecological Oncological Trial Groups; FOLFIRI, Irinotecan, calcium folinate, and fluorouracil; GC, gastric cancer; GCIG, Gynecologic Cancer Intergroup; HNSCC, head and neck squamous cell carcinoma; HSOPC, hormone sensitive oligometastatic prostate cancer; IgG1, immunoglobulin G1; IND, investigational new drug; iPSC, induced pluripotent stem cell; IV, intravenous injection; mAb, monoclonal antibody; mCRPC, metastatic castrate resistant prostate cancer; mFOLFOX6, oxaliplatin, calcium folinate, and fluorouracil; MIBC, muscle-invasive bladder cancer; MM, multiple myeloma; mPDAC, metastatic pancreatic ductal adenocarcinoma; MSS-CRC, microsatellite-stable colorectal cancer; NSCLC, non-small-cell lung cancer; OC, ovarian cancer; PC, pancreatic cancer; PCa, Prostate Cancer; PDAC, pancreatic ductal adenocarcinoma; pMMR/MSS, microsatellite stabilized; RAAA, ruptured abdominal aorta aneurysm; RAIN, Recombinant Anti-Inflammatory fusioN; SC, subcutaneous injection; SCC, squamous cell cancer; SOC, standard of care; TNBC, triple-negative breast cancer; UGTM, upper gastrointestinal tract malignancies.

### 3.2 Targeting CD73 antisense RNA

According to the gene (NIH), CD73 was encoded by NT5E. There are multiple long noncoding antisense RNAs (lncRNAs) and genes in the same region of NT5E. The same position of NT5E in human includes lncRNAs and genes in the same direction (inlcuding LOC121132697, LOC127406705, LOC127406706, LOC127406707, LOC127406708, LOC127406709, LOC129661796, LOC129996774, LOC129996775, LOC129996776, LOC129996777, and LOC129996778) and those in the opposite direction (including DUTP5, SNX14, SYNCRIP, and TPT1P6) (**Figure 1C**). The NT5E in house mouse in the same direction includes LOC131376145, LOC131376146, LOC132440797, LOC132440798, LOC132440799, and Gm10163, while the opposite direction includes miR12205, SNX14, SYNCRIP, and Gm5066. The norway rat in the same direction includes Gabarapl3 and LOC134480214, while the opposite direction includes Rps15-ps11, SNX14, and SYNCRIP. Notably, the DNA region of NT5E in humans contains both LOC129996774 and LOC129996775. The same position of NT5E in human, house mouse, and norway rat includes SNX14 and SYNCRIP. Targeting LOC129996774, LOC129996775 SNX14, and SYNCRIP may be a novel agent development strategy by regulating CD73 expression and immune homeostasis. However, the role of LOC129996774, LOC129996775, SNX14, and SYNCRIP on CD73 is unclear. In addition, the DNA region of NT5E in house mouse and norway rat did not contain LOC129996774 and LOC129996775. Notably, lncRNA NT5E (lncNT5E) was located on human chromosome 6q14.3. LncNT5E promotes pancreatic cancer (PC) development and may be a poor prognosis biomarker of PC (156). CircNT5E (also named hsa_circ_0077232), a novel circRNA derived from NT5E, promoted the development of multiple tumors, including bladder cancer (157), glioblastoma (GBM) (158), and non-small cell lung cancer (NSCLC) (159). CircNT5E promoted NT5E expression in U87 and U251 cells (158). Targeting CD73 with lncNT5E and circNT5E, specifically, circNT5E, may be a novel strategy for agent development. However, the role of lncRNA NT5E on NT5E is unclear. CircNT5E did not change NT5E expression in A549 cells (159). Research on lncNT5E and circNT5E is also scarce, with only four references in PubMed. More research is needed to confirm the feasibility of lncNT5E and circNT5E development.

## 4 Summary

Serum CD73 is a potential biomarker of diabetes and atherosclerosis. However, the selection of biomarkers should consider disease status, predisease status, or prognosis and should be more sensitive, specific, and easier to detect than existing markers. The upregulation of CD73 with agents, including AGT-5, Aire-overexpressing DCs, Aspirin, BAFFR-Fc, CD4+ peptide, ICAs, IL-2 therapies, SAgAs, sCD73, stem cells, RAD51 inhibitor, TLR9 inhibitor, and VD, decreased the development of diabetes and atherosclerosis in preclinical trials. However, the downregulation of CD73 with agents, including benzothiadiazine derivatives and CD73 siRNA, decreased atherosclerosis. ECs CD73 is an antiatherosclerotic factor, while VSMCs CD73 is a proatherosclerotic factor, suggesting that the role of CD73 in atherosclerosis may depend on its localization. However, CD73 may change from anti-atherosclerosis to pro-atherosclerosis with age. More studies were needed to confirm the role of CD73 in atherosclerosis. In addition, CD73 has a cardioprotective function in heart failure (HF) and myocardial infarction (MI). CD73 has a protective effect on the liver and kidney during ischemia-reperfusion injury (IR/I). The function of CD73 in multiple organ systems and cell types is reviewed by Minor et al. (160). Notably, many agents, including ABSK051, AK131, ATN-037, AP401, APB-A2, BB-1709, BPI-472372, BR101, BMS-986179, CBO421, Dalutrafusp alfa, Dresbuxelimab, FP-1201, ^68^GA-DOTA-dPNE, GI-108, HB0045, HB0046, HB-0052, HBM1007, HLX23, IBI325, INCA-00186, IPH5301, JAB-BX100, JAB-BX102, LY-3475070, Mupadolimab, Oleclumab, ORIC-533, PM-1015, PT199, Quemliclustat, S095024, SRF-373, and Uliledlimab, was investigated in clinical trials. However, most of these agents were CD73 inhibitors and were used to treat acute lung injury (ALI), acute respiratory distress syndrome (ARDS), cancer, and COVID-19. Only FP-1201 was a CD73 agonist and was investigated in phase 1/2/3 clinical trials. However, the further development of FP-201 was discontinued. CD39/CD73 BFP and recombinant fusion protein CD39/CD73 transgenic exosomes, the CD73 agonist were investigated for the treatment of inflammatory disease. However, more studies are needed to confirm whether clinical trials are warranted. Whether CD73 agonists are worth developing remains to be seen. In addition, many lncRNAs, circRNAs, and genes are located at the same chromosomal location as CD73. In particular, circNT5E promoted CD73 expression. circNT5E may be a promising target for agent development. However, circRNAs usually have many target genes, so how to reduce off-target effects also needs more research.

In summary, CD73 is a potential biomarker of diabetes and atherosclerosis. Targeting CD73 could improve the success rate of drug development. As research continues and technology advances, we believe that new agents will be developed to combat diseases.
